# The Effects of Situational Peers and Support Groups on the Well-Being of Grandmothers Raising Grandchildren

**DOI:** 10.1093/geroni/igaa057.1316

**Published:** 2020-12-16

**Authors:** Alexandra Jeanblanc, Carol Musil, Christopher Burant, McKenzie Wallace

**Affiliations:** 1 Case Western Reserve University, Cleveland, Ohio, United States; 2 Case Western Reserve University, Parma, Ohio, United States; 3 Case Western Reserve University, Lorain, Ohio, United States

## Abstract

Grandmothers raising their grandchildren face not only the demands of parenting, but the added burden of parenting a child they did not expect to raise. Similarly, grandmothers living in multigenerational households need to balance expectations and household/caregiving tasks across the generations. As part of a nationwide RCT designed to lessen the stress associated with the caregiving burden of raising grandchildren, we asked 342 grandmothers raising their grandchildren about their engagement with support groups and whether their social network included other grandfamilies. Here, we examine the effect of situational peers and support group engagement on grandmother’s stress, reward, social support, mental health, mindfulness, and resourcefulness. Of our sample, 53.8% (N=184) reported belonging to a support group. The majority of participants (138) belonged to online support groups on Facebook, 41 reported participating in in-person support groups, 3 belonged to both in-person and online support groups, and 8 did not respond. When asked whether and how well our participants knew other families like theirs, 31% (106) said they did not know any, 40.6% (139) knew of at least one, but not well, and 28.4% (97) said they had at least one friend with a family like her own. Knowing other families like their own affected self-appraised stress, but no other outcomes. Grandmothers participating in support groups had lower mindfulness scores, higher stress, and worse mental health scores than grandmothers not participating in support groups, possibly reflecting the higher need for support among those participants.

